# The Patients’ Beliefs Regarding the Role of Food, Mucosal Trauma, Menstruation, and Psychological Stress in the Recurrence of Behçet’s Disease Symptoms

**DOI:** 10.25122/jml-2019-0153

**Published:** 2020

**Authors:** Saeedeh Shenavandeh, Marziye Asis, Mohammad Hassan Eftekhari, Elham Aflaki, Gholam Reza Abdollahifard, Marjan Anvar Abnavi, Afsaneh Ahmadi

**Affiliations:** 1. Department of Internal Medicine, Division of Rheumatology, Shiraz University of Medical Sciences, Shiraz, Iran; 2. Department of Clinical Nutrition, School of Nutrition and Food Sciences, Shiraz University of Medical Sciences, Shiraz, Iran; 3. Department of Community Medicine, Shiraz University of Medical Sciences, Shiraz, Iran; 4. Department of Psychiatry, Shiraz University of Medical Sciences, Shiraz, Iran; 5. Research Center for Health Sciences, Department of Nutrition, School of Nutrition and Food Sciences, Shiraz University of Medical Sciences, Shiraz, Iran

**Keywords:** Behçet’s disease, diet, mucosal injury

## Abstract

Behçet’s disease is a systemic vasculitis. Mucocutaneous involvement is the most prominent finding, but triggering factors are not well-known. We decided to assess the beliefs of patients with Behçet’s disease regarding the potential role of food, mucosal injury, menstruation, and stress in the appearance of symptoms. In this cross-sectional study, 60 patients with Behçet’s disease who fulfilled the International Study Group criteria for Behçet’s disease and referred to the outpatient Behçet’s clinic of Motahari, affiliated to Shiraz University of Medical Sciences, were included. A questionnaire was designed by the research team consisting of the rheumatologist involved in the study, two dietitians, and a psychiatrist. The patients were interviewed face-to-face to fill in the questionnaire. The assessed variables were all food categories, menstruation, psychological stress, and oral mucosal injury as the potential triggers of symptoms onset. The most common foods reported as triggers for oral ulcers were eggplant (78.3%), melon (68.3%), walnut (68.2%), and cantaloupe (66.7%). Walnut was reported by three patients (5%) as the most common trigger for genital ulcers. Nervous tension (83%) and annoying arguments (45%) were the two most common psychological stress triggers for oral ulcers. Seven patients (11.7%) reported tooth brushing, as the trigger for oral ulcers. The irregular menstrual cycle was a trigger for oral ulcers in only two patients. Food items such as eggplant, walnut and melon were common self-reported triggers for mucocutaneous lesions in patients with Behçet’s disease. Nervous tension and annoying arguments were also common psychological triggers for oral aphthous ulcers.

## Introduction

Behçet’s disease (BD) is a multi-systemic immune-mediated vasculitis [1]. BD has a wide array of manifestations, including oral and genital ulcers, ocular disease, skin lesions, gastrointestinal and neurologic involvement, and even arthritis, vascular and pulmonary manifestations [2]. The most important clinical symptoms are oral aphthous ulcers, genital ulcers, and ocular lesions (especially uveitis), which are considered as a classic triad. It is a chronic relapsing condition with varying degrees of severity in each acute inflammatory relapse [3]. Recurrent oral aphthous ulcers (stomatitis) are usually the most common initial manifestations of the disease that causes the clinician to suspect the diagnosis [4].

BD is more prevalent in some geographical regions (ancient Silk Road), including Turkey, Iran, the Mediterranean region, and East Asia [5], with an estimated prevalence of 14 to 20 per 100,000 persons [1]. However, BD is much less prevalent in North America and western European countries (6). The exact reasons for acute inflammatory attacks and respective severity and duration are still not understood completely, and the attacks do not follow a predictable pattern [3].

As acute inflammatory attacks are unpredictable, efforts have been made to find exogenous factors that can make the role of triggers for BD acute attacks of inflammation. The factors that have been mentioned in the literature as potential triggers include food [7], psychological stress [8], mucosal trauma, menstruation [9], dental extractions, and infections [7]. It has been shown that surgery or minimally invasive procedures in patients with Behçet’s syndrome can be a trigger for its vascular events [10]. The results of the mentioned studies have indicated that the potential triggers have been reported and recalled by the patients. However, no consistent data are supporting these findings and given the fact that these factors can be avoided and, therefore, decrease the rate of recurrent inflammation attacks in BD, it seems that more studies are required to address this issue.

In this study, we decided to determine the role of potential triggers for BD manifestations development/progression, especially oral and genital ulcers and ocular lesions.

## Material and Methods

In this cross-sectional study, the study population consisted of BD patients who fulfilled the International Study Group criteria on Behcet’s disease (ISG criteria, 1990). The study lasted for one year (from 2016 to 2017). The included patients were those referred to our outpatient Behçet’s clinic of Motahari, affiliated with Shiraz University of Medical Sciences, Shiraz, Iran. All patients with BD who had medical records at our clinic were eligible to enter the study voluntarily. A rheumatologist involved in this study included the patients randomly and conducted face-to-face-questionnaires. The questionnaire was designed by the research team consisting of rheumatologists, two dietitians, and a psychiatrist who examined literature reviews and had several meetings to include the factors for which evidence is available as potential triggers for BD acute inflammatory attacks. It included factors like all food categories, menstruation, psychological stress, and mucosal trauma. All food categories were included in the questionnaire by consulting two dietitians. The food categories were fruits, vegetables, nuts and dried fruits, drinks, pastry and sugary foods, meat and egg and fish, milk and dairy products, and honey. Psychological stress domains were nervous tension, annoying arguments, painful life events, apprehension about the future, sleep deprivation, and public speaking. Tooth brushing was considered when studying traumatic injuries of the oral mucosa.

The questionnaire was then reviewed independently by three rheumatologists for accuracy and validity. In order to test the reliability of the questionnaire, 30 patients were asked to complete the questionnaire. After two weeks, the patients were asked to fill out the questionnaire again for the second time. The Cronbach’s alpha was calculated as 0.6, so the reliability was confirmed.

Also, demographic data, including gender, age, BD duration, occupation, marital status, residential place, educational level, and lifestyle (sedentary or active), were gathered. The patients were personally interviewed after being referred to the clinic for routine follow-up in the clinic of Behçet’s disease.

### Statistical analysis

Descriptive statistics such as frequency, percentage, mean, and standard deviation (SD) were used to express the data. The analyses were done using the SPSS software (ver. 20.0, IBM, USA).

## Results

A total of 60 patients with BD were enrolled in the study and completed the questionnaires. The mean (±SD) age of the participants was 43.48 (± 1.72) years. Mean (±SD) BD time passed from the diagnosis was 13.16 (± 1.01) years. Table 1 presents demographic data of the studied patients.

**Table 1:** Demographic data of 60 patients with Behçet’s disease.

		**Frequency**	**Percentage**
**Gender**	**Male**	27	45%
	**Female**	33	55%
**Marital status**	**Married**	54	90%
	**Single**	6	10%
**Occupation**	**Practitioner**	33	55%
	**Housewife**	26	43.33%
	**Unemployed**	1	1.67%
**Residential place**	**Urban**	45	75%
	**Rural**	15	25%
**Educational level**	**Illiterate**	3	5%
	**High school graduate**	8	13.3%
	**Primary school**	19	31.7%
	**Intermediate**	14	23.3%
	**High school**	7	11.7%
	**Some college**	3	5%
	**University degree**	6	10%
**Lifestyle**	**Sedentary**	4	6.67%
	**Active**	56	93.33%

### Food and oral aphthous ulcers

**Figure 1:**
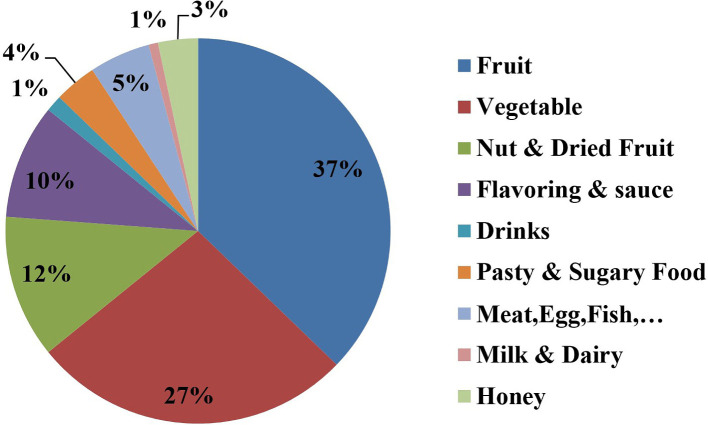
Frequency distribution of food groups mentioned by 60 patients with Behçet’s disease as triggers for oral aphthous ulcers.

Eggplant (78.23%), melon (68.3%), walnut (68.2%), and cantaloupe (66.7%) were mentioned as the most common food triggers for BD-related aphthous oral ulcer recurrences. Table 2 presents the frequency of foods mentioned by the patients as triggers for oral aphthous ulcer development. Other foods which were reported less frequently (≤ 5 patients) included chocolate, peanuts, sausages, egg, coffee, spinach, cocoa, potato, peach, watermelon, tangerine, grapes, raisins, hamburger, mayonnaise sauce, potato chips, sour lemon, yogurt, kitchen, fish, tuna fish, and meat. Other foods that had a very low frequency (only in one patient) were tea, biscuits, cookies, organ meat, red meat, sunflower seeds, mushroom, lettuce, beans, fava beans, grapefruit, lemon (chino), cherry, milk, garlic, and ice cream. Figure 1 shows the frequency distribution of food groups mentioned by the patients as triggers for oral aphthous ulcers.

**Table 2:** Foods mentioned by patients with Behçet’s disease as triggers for the development of oral aphthous ulcers.

	**Frequency**	**Percentage**
**Eggplant**	47	78.3%
**Melon**	41	68.3%
**Walnut**	41	68.3%
**Cantaloupe**	39	65%
**Pepper**	31	51.7%
**Fig**	29	48.3%
**Tomato**	29	48.3%
**Bell peppers**	20	33.3%
**Kiwi**	18	30%
**Banana**	17	28.3%
**Honey**	17	28.3%
**Pickled fruits**	12	20%
**Halva**	11	18.3%
**Pistachios**	11	18.3%
**Tomato ketchup**	7	11.7%
**Spice**	6	10%

**Figure 2:**
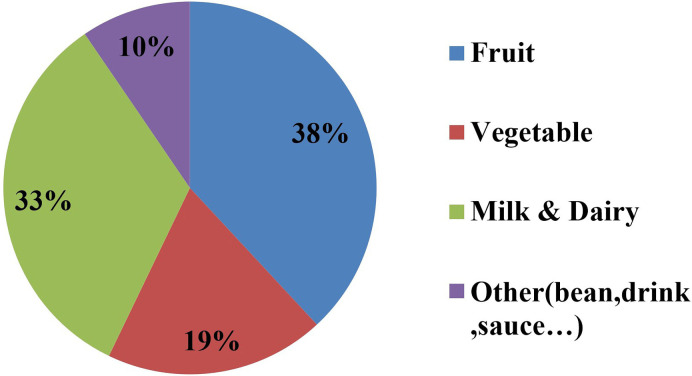
Frequency distribution of food groups mentioned by patients with Behcet’s disease as the factors that decreased the occurrence of oral aphthous ulcers.

In contrast to the foods reported by the patients as triggers of oral aphthous ulcers, other food groups were reported as factors that decreased the occurrence of oral aphthous ulcers. These included watermelon, cheese, yogurt (each was reported by three patients, 5%), milk, dough (a salty yogurt drinking), kashk (a dairy product), orange, lemon (chino), cucumber, apple, cherry, carrot, spinach, parsley, and ice cream (each was reported by two patients, 3.33%). Figure 2 shows the frequency distribution of food groups that were mentioned by the patients as the factors that decreased the occurrence of oral aphthous ulcers.

### Food and genital ulcers

Walnuts were reported by three patients (5%) as the most common triggers for the occurrence of genital ulcers. Other food triggers for this BD presentation were melon, bell peppers, peanuts, and honey (each reported by two patients, 3.33%). Other less common foods reported only by one patient were cantaloupe, eggplant, banana, pickled fruits, chocolate, pistachio, dates, tomato, pepper, and coffee.

### Food and cutaneous pustules

The foods that were described as triggers for cutaneous pustules were figs, melon, eggplant, pepper, bell pepper, walnut, chocolate, and coffee, each reported by one patient (1.67%).

### Food and ocular pain and redness (ocular inflammation)

Pepper was reported by two patients (3.33%), and coffee, eggplant, cantaloupe, melon, bell pepper, tomato, and walnuts (each reported by one patient, 1.67%) were the foods that were reported as triggers for ocular inflammation.

### Food and joint inflammation(swelling and pain)

None of the foods in the questionnaire was reported by the patient as a trigger for joint inflammation.

### Psychological stress

Five domains were included in the questionnaire as indices for psychological stress. Table 3 presents the frequency distribution of the five domains as triggers for BD symptoms. The nervous tension was the most common psychological stress as a trigger for BD manifestations. About 83% of the patients mentioned nervous tension as the trigger for oral aphthous ulcers (Table 3).

**Table 3:** Frequency distribution of six domains of psychological stress as triggers for Behcet’s disease manifestations.

	**Trigger for oral aphthous ulcers**	**Trigger for genital ulcers**	**Trigger for skin lesions**	**Trigger for ocular inflammation**	**Trigger for joint inflammation**
**Nervous tension**	50 (83.3%)	3 (5%)	4 (6.7%)	2 (3.33%)	2 (3.33%)
**Annoying argument**	27 (45%)	1 (1.67%)	2 (3.33%)	0	0
**Painful life events**	23 (38.3%)	0	3 (5%)	0	1 (1.67%)
**Apprehension about the future**	23 (38.3%)	1 (1.67%)	1 (1.67%)	0	0
**Sleep deprivation**	20 (33.3%)	0	3 (5%)	1 (1.67%)	0
**Public speaking**	4 (6.7%)	0	0	0	0

### Mucosal trauma

Seven patients (11.7%) reported tooth brushing as the trigger for oral ulcers. However, 14 subjects mentioned tooth brushing as the factor that decreased the occurrence of oral ulcers. One patient reported oral ulcers in the case of using mint toothpaste or chewing mint gums. One patient also reported oral ulcers when using mint toothpaste.

### Menstruation

Of the 60 patients, 33 were female. Eleven patients were in the post-menopausal state. Of the remaining 22 female patients, 17 cases had regular menstrual cycles, and 5 had irregular menstrual cycles. In the case of irregular menstrual cycles, two patients (6.1%) experienced oral ulcers, one (3%) reported an increase in genital ulcers and one (3%) reported pustular skin lesion recurrences. Also, two patients (6.1%) reported the recurrence of oral ulcers when taking oral contraceptive pills, and one patient mentioned a genital ulcer at the end of her pregnancy.

## Discussion

Based on the obtained findings, several food items were reported by a significant number of patients with BD as triggers for oral and genital ulcers. Some components of psychological stress, including nervous tension and annoying arguments, were also mentioned as triggers for oral and genital ulcers. Despite the significance of food items and psychological stress, oral mucosal trauma inflicted by tooth brushing and menstruation irregularities were not reported as common triggers of BD symptoms.

There are few studies about the role of diet and foods on the progression/development of BD symptoms. In a recent study addressing dietary triggers of oral ulcers in BD [7], the authors reported that food was recognized as triggers by about one-third of the patients surveyed. Nuts, pineapple, and peanuts were the most frequent triggers reported for oral ulcers. Here, we also found that walnut was the third most commonly reported trigger for oral ulcers. However, eggplant and melon were more commonly reported than walnuts. We also assessed not only oral ulcers but also genital ulcers and pustular skin lesions. Walnut, eggplant, and melon were also recognized as common triggers for genital ulcers and skin lesions. Based on these findings, it appears that mucocutaneous lesions studied had common food triggers. In justifying the food triggers, histamine, which is a proinflammatory mediator, has been reported to be high in eggplant. In fact, high histamine level in eggplants has been suggested as the cause of the high rate of allergy to eggplant [11]. Eggplant is a vegetable that is commonly used in Iranian cuisine. Non-protein components of eggplant have gained more attention than protein allergens, and allergy to eggplant had been reported to have a relatively high prevalence, involving 10% of the general population [12]. Nuts are also well known for causing allergic reactions [13].

More important than food items, psychological stress items, especially nervous tension, annoying arguments, and painful life events, were reported by a considerable number of patients as triggers for oral and genital ulcers. This finding is comparable to previous studies [7, 8]. In one study, similar to what we observed in our study, 79% of the patients reported the occurrence of the disease by stressful life events [8].

Menstrual abnormalities/irregularities and oral mucosa trauma were not reported as prevalent triggers for BD. In a similar study, oral mucosal trauma (biting) was only reported by 2% of the surveyed patients [7]. In the mentioned study, similar to our findings, menstruation was only reported by 4% of the patients as a significant trigger. These findings contradict the results of a recent study that revealed acneiform lesions exacerbation in about two-thirds of BD patients related to menstruation (14).

As predicted, oral ulcers were the most common reported symptoms by the studied patients. It is well established that oral ulcers are associated with unfavorable quality of life in BD patients [4]. Considering this fact, the findings presented here can be used for future studies to investigate the effect of the suggested triggers in avoiding or decreasing the rate of BD symptoms. This requires longitudinal studies to elaborate on the real effect of dietary interventions and psychological consultations in decreasing the occurrence of symptoms or their severity.

However, we faced some limitations in this study. Firstly, the nature of the study was subjective, and recall bias likely affects the responses the patients provided. In addition, we were not able to analyze the results as the study was descriptive and, therefore, we only provided descriptive statistics, and we cannot conclude a causative relationship between the triggers and BD symptoms.

## Conclusion

Food items such as eggplant, walnut, and melon were common self-reported triggers for mucocutaneous lesions in BD patients. Nervous tension and annoying arguments were also common psychological triggers for oral ulcers in BD patients. However, oral mucosal trauma and menstrual irregularities were not commonly found to be food and psychological stress triggers. These findings can be used for further clinical trials to study the mentioned triggers as potential interventional options in BD.

## Conflict of Interest

The authors declare that there is no conflict of interest.

## Acknowledgments

The present article was extracted from the thesis written by Dr. Marzyie Asis, supported by the Shiraz University of Medical Science (Grant no: 92-01-01-5778). The authors would like to thank the Shiraz University of Medical Sciences, Shiraz, Iran, and Center for Development of Clinical Research of Nemazee Hospital, as well as Dr. Nasrin Shokrpour for editorial assistance.
